# Complete Genome Sequence of Mycobacterium sp. Strain VKM Ac-1817D, Capable of Producing 9α-Hydroxy-androst-4-ene-3,17-dione from Phytosterol

**DOI:** 10.1128/genomeA.01447-14

**Published:** 2015-01-29

**Authors:** Victoriya Y. Shtratnikova, Mikhail I. Schelkunov, Dmitry V. Dovbnya, Yury A. Pekov, Eugeny Y. Bragin, Vasily V. Ashapkin, Marina V. Donova

**Affiliations:** a Department of Bioengineering and Bioinformatics, M.V. Lomonosov Moscow State University, Moscow, Russia; b G.K. Skryabin Institute of Biochemistry and Physiology of Microorganisms, Russian Academy of Sciences, Pushchino, Moscow Region, Russia; c A.N. Belozersky Institute of Physico-Chemical Biology, M.V. Lomonosov Moscow State University, Moscow, Russia

## Abstract

Mycobacterium sp. strain VKM Ac-1817D is capable of converting phytosterol into 9α-hydroxy androst-4-ene-3,17-dione (9-OH-AD), which is a valuable intermediate for the steroid pharmaceutical industry. Here, a complete genome sequence of the strain is reported. The genome consists of a single circular 6,324,222-bp chromosome with a G+C content of 66.2% and encodes approximately 6,000 CDSs, 54 tRNAs, and 6 rRNAs.

## GENOME ANNOUNCEMENT

The fast-growing nonpathogenic mycobacteria were reported to carry out side-chain degradation of natural sterols, such as phytosterol, to form 3-keto-4-ene androstanes, which are the key intermediates for the synthesis of pharmaceutical steroids (1). Mycobacterium sp. strain VKM Ac-1817D effectively generates 9α-hydroxy androst-4-ene-3,17-dione (9-OH-AD) as a major product from phytosterol. Recently, on the basis of genome data mining and the assembly of contigs, we evaluated the strain features that are of significance for phytosterol catabolism (2). In this work, we report the complete genome assembly.

A short-read library containing DNA fragments of 364 ± 17 bp insert length was prepared with a DNA sample preparation kit (New England Biolabs) after digestion of genomic DNA with NEBNext dsDNA Fragmentase. The library was sequenced on Genome Analyzer IIx (paired-end 72-nucleotide reads) and HiSeq 2000 (paired-end 100-nucleotide reads). The mate-pair library with fragments of 3,720 ± 1918 bp was created with the Nextera mate-pair sample preparation kit (Illumina) and was also sequenced on the HiSeq 2000 platform. NextClip version 0.8 (3) was used to remove paired-end contamination in the mate-pair reads. The reads were adapter and quality trimmed by Trimmomatic 0.32 (4). *De novo* genome assembly was performed with Velvet version 1.2 (5), Spades version 3.1.0 (6), and CLC Genomics Workbench version 6.0 (http://www.clcbio.com) using both paired-end and mate-pair reads. The contigs produced by the assemblies were manually combined into a single circular contig in BioEdit (7). Quality of the resulting contig was assessed by REAPR version 1.0.17 (8). The contig was also checked by mapping reads in CLC Genomics Workbench and visually inspecting putatively ambiguous places. Mean coverage of the resulting genome by two libraries was 621.

The genome is 6,324,222 nucleotides in length, with a GC content of 66.2%. Annotation of the genome was carried out both with RAST (9) and NCBI Prokaryotic Genome Annotation Pipeline (PGAP; http://www.ncbi.nlm.nih.gov/genome/annotation_prok). The RAST annotation revealed 6,118 CDSs, and the annotation by PGAP revealed 6,043 genes, of which 5,691 were determined to be protein-coding genes and 291 to be pseudogenes. The genome encodes 54 tRNAs (of which one is pseudo and 43 of the other 53 are unique), 6 rRNAs, and 1 ncRNA.

The complete genome sequence is useful for genome-based approaches to genetic manipulations for the purpose of creating novel sterol-transforming biocatalysts for the steroid industry.

### Nucleotide sequence accession number.

The complete genome sequence has been deposited in GenBank under the accession number CP009914.

## References

[1] Donova MV, Egorova OV 2012 Microbial steroid transformations: current state and prospects. Appl Microbiol Biotechnol 94:1423–1447. doi:10.1007/s00253-012-4078-0. 22562163

[2] Bragin EY, Shtratnikova VY, Dovbnya DV, Schelkunov MI, Pekov YA, Malakho SG, Egorova OV, Ivashina TV, Sokolov SL, Ashapkin VV, Donova MV 2013 Comparative analysis of genes encoding key steroid core oxidation enzymes in fast-growing *Mycobacterium* spp. strains. J Steroid Biochem Mol Biol 138:41–53. doi:10.1016/j.jsbmb.2013.02.016. 23474435

[3] Leggett RM, Clavijo BJ, Clissold L, Clark MD, Caccamo M 2014 NextClip: an analysis and read preparation tool for Nextera Long Mate Pair libraries. BioInformatics 30:566–568. doi:10.1093/bioinformatics/btt702. 24297520 PMC3928519

[4] Bolger AM, Lohse M, Usadel B 2014 Trimmomatic: a flexible trimmer for Illumina sequence data. BioInformatics 30:2114–2120. doi:10.1093/bioinformatics/btu170. 24695404 PMC4103590

[5] Zerbino DR, Birney E 2008 Velvet: algorithms for *de novo* short read assembly using de Bruijn graphs. Genome Res 18:821–829. doi:10.1101/gr.074492.107. 18349386 PMC2336801

[6] Bankevich A, Nurk S, Antipov D, Gurevich AA, Dvorkin M, Kulikov AS, Lesin VM, Nikolenko SI, Pham S, Prjibelski AD, Pyshkin AV, Sirotkin AV, Vyahhi N, Tesler G, Alekseyev MA, Pevzner PA 2012 SPAdes: a new genome assembly algorithm and its applications to single-cell sequencing. J Comput Biol 19:455–477. doi:10.1089/cmb.2012.0021. 22506599 PMC3342519

[7] Hall TA 1999 BioEdit: a user-friendly biological sequence alignment editor and analysis program for Windows 95/98/NT. Nucleus Acids Symp Ser 41:95–98.

[8] Hunt M, Kikuchi T, Sanders M, Newbold C, Berriman M, Otto TD 2013 REAPR: a universal tool for genome assembly evaluation. Genome Biol 14:R47. doi:10.1186/gb-2013-14-5-r47. 23710727 PMC3798757

[9] Aziz RK, Bartels D, Best AA, DeJongh M, Disz T, Edwards RA, Formsma K, Gerdes S, Glass EM, Kubal M, Meyer F, Olsen GJ, Olson R, Osterman AL, Overbeek RA, McNeil LK, Paarmann D, Paczian T, Parrello B, Pusch GD, Reich C, Stevens R, Vassieva O, Vonstein V, Wilke A, Zagnitko O 2008 The RAST server: rapid annotations using subsystems technology. BMC Genomics 9:75. doi:10.1186/1471-2164-9-75. 18261238 PMC2265698

